# Continuous Similarity Learning with Shared Neural Semantic Representation for Joint Event Detection and Evolution

**DOI:** 10.1155/2020/8859407

**Published:** 2020-12-12

**Authors:** Pengpeng Zhou, Yao Luo, Nianwen Ning, Zhen Cao, Bingjing Jia, Bin Wu

**Affiliations:** ^1^Beijing Key Lab of Intelligent Telecommunication Software and Multimedia, Beijing University of Posts and Telecommunications, Beijing, China; ^2^Rice University, Houston, TX, USA

## Abstract

In the era of the rapid development of today's Internet, people often feel overwhelmed by vast official news streams or unofficial self-media tweets. To help people obtain the news topics they care about, there is a growing need for systems that can extract important events from this amount of data and construct the evolution procedure of events logically into a story. Most existing methods treat event detection and evolution as two independent subtasks under an integrated pipeline setting. However, the interdependence between these two subtasks is often ignored, which leads to a biased propagation. Besides, due to the limitations of news documents' semantic representation, the performance of event detection and evolution is still limited. To tackle these problems, we propose a Joint Event Detection and Evolution (JEDE) model, to detect events and discover the event evolution relationships from news streams in this paper. Specifically, the proposed JEDE model is built upon the Siamese network, which first introduces the bidirectional GRU attention network to learn the vector-based semantic representation for news documents shared across two subtask networks. Then, two continuous similarity metrics are learned using stacked neural networks to judge whether two news documents are related to the same event or two events are related to the same story. Furthermore, due to the limited available dataset with ground truths, we make efforts to construct a new dataset, named EDENS, which contains valid labels of events and stories. The experimental results on this newly created dataset demonstrate that, thanks to the shared representation and joint training, the proposed model consistently achieves significant improvements over the baseline methods.

## 1. Introduction

In recent years, with the great development of the Internet and AI technologies, tremendous volumes of news articles to report the breaking events are being rapidly generated by various media providers, for example, TV broadcast, the governmental news website, and information Portals (sina.com, Tencent News, Headlines Today, CNN, BBC, etc.). Besides, some popular social media, such as Twitter, Sina Weibo, and Facebook, also has become an attractive platform for people for expressing opinions, broadcasting news, and discussing topics. Compared with the former traditional news media, the tweet stream produced by the emerging social media or self-media often contains many informal expressions or irrelevant information. Despite massive information about a number of ongoing hot events is spread at every moment, people still feel unable to acquire useful information about the events of concern. In solving the problem of information overload, event detection and evolution on news streams have drawn extensive research attention over the past few years [1–6]. Generally, an event refers to a particular thing that happens at a specific time and place [7], for example, Opening Ceremony of the 29^th^ Modern Summer Olympic Games held in Beijing on August 8^th^, 2008. Under the premise of this definition, event detection aims to identify a group of news documents that report the same topic possibly in different ways [8]. Therefore, a new topic can be detected if new news documents are found not belonging to existing topic clusters. Followed by event detection, event evolution connects related events logically to tell evolving stories. Event detection and evolution provide an emerging and more natural alternative way to present news corpora; hence, people can be easier to know the development process of topics of interest. In this paper, we focus on the detection and evolution of events from formal news media.

For decades, research has introduced many methods for event detection [5, 6, 9] and event evolution [1–4, 8] in the area of Topic Detection and Tracking (TDT) [10]. Of these methods, a similar pipeline is followed; that is, event detection and evolution are considered as two independent subtasks. First, news documents are aggregated into event clusters through any clustering algorithm such as *K*-means [11], incremental clustering [12], and locality sensitive hashing (LSH) [13]. In addition, there are some methods based on topic modeling [14] and term weighting [15] for event detection. After detecting extensive and diverse events, various methods such as event timelines [16], event threads [1], event evolution graphs [3], and information maps [17] are used to identify the relationship between events and present a clear story development process.

Despite great progress in these above methods, the interdependence between the event detection and evolution tasks is often ignored and has not been fully utilized. For example, a news document that comprehensively indicates the events in event detection should be scored high in the corresponding story architecture. Furthermore, as the base of events, the similarity of the two news documents also depicts the similarity of the events that documents belong to; hence, a better understanding of a news document is helpful for both event detection and event evolution. Besides, some of the popular clustering methods, for example, K-means, which requires setting the number of clusters in advance to discover events, may also not be applicable for the rapidly varying news streams that occur daily in the real world.

**Figure 1:**
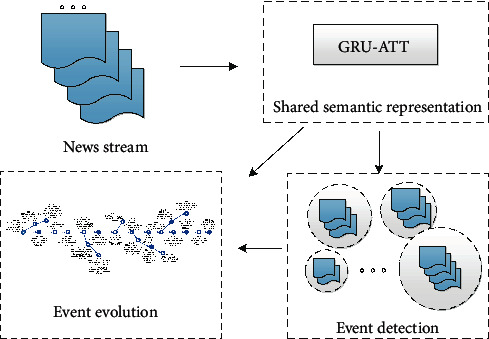
The overall architecture of our model. (1) The top left corner shows the input of our model, which is news stream consisting of a lot of news reports. (2) The top right corner shows the shared semantic representation of news stream, which is the input of both event detection network and event evolution network. Note that we utilize a standard bidirectional gated recurrent unit (GRU) model with attention mechanism to learn the shared representation across two subtasks (event detection subtask and event evolution subtask). (3) The bottom right corner shows the event detection module, in which our goal is to aggregate news reports related to the same event together. (4) After detecting a series of events, we further organize these events into multiple stories in an online manner, which forms the event evolution module in the bottom left corner.

To handle these challenges, in this paper, we investigate a neural network model that monitors the news streams in the open domain, jointly detecting new events and tracking the evolution process of events, as illustrated in Figure 1. To better describe the news documents, we introduce the advanced bidirectional GRU network followed by attention operations to achieve the fix-length vectorial semantic representation. Such a representation can depict both fine-grained news documents and holistic events; hence, it is shared across the event detection and evolution tasks. Moreover, inspired by great achievements achieved by multitask learning, we propose to jointly learn two continuous neural similarity metrics for judging whether two news documents are related to the same event or two events are related to the same story under the framework of the Siamese network. During the testing, the online incremental clustering method of news documents and events are applied for event detection and evolution, respectively. To further promote the interaction between the two subtasks, the strategy of neural stacking [18] is applied to the pipeline. Specifically, the hidden neural layers of the event detection model are fed as additional input features to the event evolution model, and the errors of event evolution are propagated to event detection during training, so that information is better shared between the predecessor detection and successor evolution.

The contributions of this paper could be summarized as follows.

- To the best of our knowledge, this is the first work that considers the correlation of event detection and event evolution and investigates a neural model for Joint Event Detection and Evolution, which benefits both the tasks.
- The introduced GRU attention network produces a more powerful shared semantic representation than traditional features as the basis of two subtasks. Furthermore, the continuous similarity metric learning from data provides a direct, effective, and robust criterion to calculate the similarity of document pairs in event detection and event pairs in event evolution, respectively. The joint learning of shared semantic representation and continuous similarity metrics provides a good foundation for later incremental clustering based event detection and evolution pipeline.
- Due to the limited available datasets with ground truths, we manually annotate a new dataset, named EDENS (Event Detection and Evolution from News Streams), in which the number of stories is 12, the number of events is 694, and each story contains 58 events on average. The dataset is freely available to the community upon request. Experimental results on the real-world EDENS dataset demonstrate that our model significantly outperforms several baseline methods in both event detection and evolution benchmarks.

The remainder of this paper is organized as follows: in Section 2, the related literature is reviewed. In Section 3, some concepts and problems are defined. In Section 4, we elaborate on the proposed joint model for event detection and evolution. Experiments and analysis are presented in Section 5. Finally, we conclude the paper and talk about future work in Section 6.

## 2. Related Work

In this section, some related literature about event detection, event evolution, and joint model is reviewed.

### 2.1. Event Detection

Following the original Topic Detection and Tracking (TDT) program [10], event detection aims to discover new or previously unidentified events from the information stream. Most of the works detect events of interest from news media [8, 11, 19] or social media [5, 20, 21]. In addition, some research works are also called event detection but are essentially different from the former task; for example, [22] talks about ACE event detection task which focuses on extracting events with entities from sentences and [23] considers the event detection as a text classification problem, that is, categorizing each event to predefined types. There are also some works concerning the detection of certain types of events, such as violent and disaster events [5, 24], significant events in the calendar [25]. In this paper, we study the problem of detecting events in the open domain from the news streams in an unsupervised clustering manner. The types of events are not specially restricted.

In general, the work on event detection can be divided into three categories, that is, word and phrase-based approaches, distance-based clustering approaches, and probabilistic model-based approaches. Specifically, word and phrase-based approaches detect events by utilizing important words or phrases. Zhou et al. [11] utilize Jaccard similarity coefficient × Inverse Dimension Frequency with time order to identify words with salient scores as event words and then extract the document embedding by applying word2vec to event words, and, finally, Bikmeans is employed to cluster all news documents based on obtained document embeddings. Slonim and Tishby [26] propose a two-phase strategy for document clustering. They first find word-clusters such that most of the mutual information between words and documents is preserved and then leverage the word-clusters to perform document clustering.

Word and phrase-based approaches mainly focus on mining event words and do not directly utilize the event-related documents. On the contrary, distance-based clustering approaches group documents into events by measuring the similarity between documents with some distance metrics like Euclidean distance or Cosine distance. The performance of clustering mainly relies on document feature representations and clustering methods. The common feature representations include TF-IDF [27, 28], BM25 term weighting [29], and neural vector [5, 20]. The clustering methods include *K*-means [30], density-based algorithms [31], incremental clustering [5, 20, 27], or self-organizing map clustering [28]. Among these approaches, those based on neural representation and online incremental clustering show the best performance in both accuracy and efficiency; hence, the proposed model in this paper also follows this strategy.

Probabilistic model-based approaches assume a document generation process and then infer topic distributions of documents by optimizing generation probability [32, 33]. Some representative topic models include Latent Dirichlet Allocation (LDA) [14], Probabilistic Latent Semantic Indexing (PLSA) [34], and Gaussian Mixture Model (GMM) [35]. These models are computationally intensive and do not produce satisfying results for a finer clustering.

### 2.2. Event Evolution

Traditional Topic Detection and Tracking (TDT) program [10] only detects new events or tracks previously spotted events, but the relationship between events is not interpreted. To help users better know the developing structure of events, different approaches have been proposed from various aspects. Reference [2] proposes a topic evolution model by discovering the temporal pattern of events with timestamps of the text stream. Reference [4] further proposes to construct a temporal event graph to analyze event evolution and determines the dependencies of two events by considering their temporal relationships, content dependencies, and event causality. Reference [1] proposes the concept of Event Threading and tries to append each new event to its most similar earlier event. The similarity between two events is measured by the TF-IDF cosine similarity of the event centroids. Reference [3] proposes to measure the similarity relationship of events by analyzing content similarity, time proximity, and document distribution and models the event evolution structure by a directed acyclic graph (DAG).

Although the above structures effectively describe the evolution process of events, sorting events by timestamps omits the logical connection between events, while graph structures do not consider the evolving consistency of the whole story, leading to unnecessary connections between events. To solve these problems, [17] proposes the Metro Map model, which defines some metrics such as coherence and diversity for story quality evaluation and identifies the storyline by solving an optimization problem to maximize the topic diversity of storylines while guaranteeing the coherence of each storyline. Reference [36] further proposes a cross-modal solution, where two requisite properties of an ideal storyline, that is, coherence and diversity, are investigated; then, a unified algorithm is devised to extract all effective storylines by optimizing these properties at the same time. These works regard the event evolution as an optimization problem with given news corpora. However, they do not deal with newly generated documents at any time and update story structures in an online manner. Recently, [8] proposes a structure of story tree to characterize the evolution of the story, grows story trees with events online, and presents the most effective representation effect according to extensive user experience. In this paper, various evolution structures are investigated in conjunction with deep learning based event clustering algorithm.

### 2.3. Neural Joint Modeling

The joint model has been extensively researched in various NLP tasks, for example, joint entity and relation extraction [37], joint event extraction and visualization [38], joint event detection and summarization [5, 39], joint event detection and prediction [23], and joint parsing and name entity recognition [40]. The key to joint models is designing shared features to capture mutual information between the integrated tasks. For this purpose, neural network models have been shown effective by inducing semantic features automatically after data-driven joint training [5, 41].

Among most of the neural joint models, two main strategies have been employed. On one hand, shared semantic representation learning between different tasks is crucial; for example, a shared LSTM layer is used for joint event detection and summarization [5], as well as joint event detection and prediction [23]. On the other hand, neural stacking is also widely applied by feeding the hidden layer of a predecessor model as additional input features to its successor model [5, 18, 42]. During training, errors of the predecessor model can thus be propagated to its successor model, so that information is better shared between the predecessor and successor models. In this paper, we first investigate the joint neural model for event detection and event evolution through a shared semantic representation and stacking model.

## 3. Preliminary

In this section, we first describe some key concepts and notations used in this paper and formally define our problem.

### 3.1. News Report

A news report (often edited by journalists) is usually to report significant real-world breaking news. Different from popular tweets in social media, which contain various expressions of the same meaning, many informal colloquial phrases, or irrelevant information, most news reports formally describe time, location, person, organization, action, and some important keywords related to the news. In this paper, a news report *R*_*i*_ is represented as *R*_*i*_=(*d*_*i*_, *τ*_*i*_, *t*_*i*_), where *d*_*i*_, *τ*_*i*_, and *t*_*i*_ are the news body, news title, and news publish time, respectively.

### 3.2. News Stream

A news stream is a continuous and temporal stream of news reports {*R*_1_, *R*_2_,…, *R*_*k*_,…} starting at an initial time *t*_0_.

### 3.3. Event

An event *E* is a particular thing that happens at a specific time and place [7, 43]. Specifically, it is a set of news documents reporting the same real-world breaking news [8].

### 3.4. Event Timestamp

As mentioned before, an event consists of several news reports, and each report may have a different publish time. In order to clearly suggest the temporal information of an event, we simply consider the earliest news report time as the event timestamp.

### 3.5. Story

A story *S* describes a news topic and comprises a set of related events that report a series of evolving real-world breaking news [8]. A directed link from events *E*_1_ to *E*_2_ indicates a temporal development or a logical connection between two events. An example can help further clarify the concept of stories versus events; that is, “The ZTE incident” in Table 1 is a story, which consists of several events, such as “2018-04-17: the US government bans ZTE from buying sensitive products from the US companies,” “2018-04-19: ZTE issues an internal letter and sets up a crisis task force to urge employees to be clear-headed,” “2018-04-23: ZTE issues a further announcement: measures have been taken to comply with the rejection order,” …, “2018-06-08: ZTE's ban is lifted and $1 billion is fined,” …, “the ZTE incident is over, and ZTE will start again with confidence.”

### 3.6. Event Detection

Following the Topic Detection and Tracking (TDT) program [10], the task of event detection (or event clustering) is to cluster news reports of the same real-world events, so that a new event can be detected if new news reports are found not belonging to existing event clusters.

### 3.7. Event Evolution

The task of event evolution is to connect the extracted related events to form a story, that is, *S*={*E*_1_, *E*_2_,…, *E*_*n*_}. Since the relationship of event evolution includes temporal relationship or logical relationship [8], we can describe event evolution from different aspects in this paper.

### 3.8. Joint Event Detection and Evolution

Given a news stream *R*={*R*_1_, *R*_2_,…, *R*_*k*_,…}, where *R*_*i*_=(*d*_*i*_, *τ*_*i*_, *t*_*i*_) represents the news report published at time *t*_*i*_, our objective is to jointly cluster all news reports *R* into a set of events *E*={*E*_1_, *E*_2_,…, *E*_|*E*|_} and connect the extracted events to form a set of stories *S*={*S*_1_, *S*_2_,…, *S*_|*S*|_}, where each story *S*_*i*_={*E*_*i*_1__, *E*_*i*_2__,…, *E*_*i*_*n*__} contains a set of related events *E* of the same topic.

## 4. Joint Model for Event Detection and Evolution

**Figure 2:**
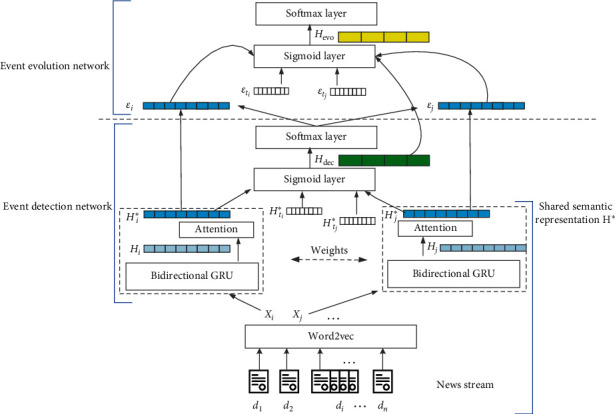
Illustration of our JEDE model. JEDE consists of two submodels: event detection network and event evolution network. They are based on the shared semantic representation and additional temporal information. The submodels are stacked to enable information sharing between the two tasks.

In this section, we elaborate on the proposed neural model for Joint Event Detection and Evolution (JEDE). The overall framework is illustrated in Figure 2, where *H*^*∗*^ is the shared semantic representation of a news report document, *H*_dec_ is the hidden state of event detection, and *H*_evo_ is the hidden state of event evolution. Specifically, the proposed JEDE model consists of two submodels: event detection network and event evolution network, which are based on the shared semantic representation. Besides, two submodels are stacked by feeding the hidden layer of the predecessor event detection network as additional input features to its successor event evolution network. The above design brings two advantages. On the one hand, as the common input of two subtasks, the shared semantic representation can achieve the optimal parameters through joint training across tasks, which benefit both subtasks. On the other hand, the neural stacking manner makes the errors of event evolution be propagated to event detection during the training stage, so that information is better shared between the predecessor event detection and successor event evolution. Hence, the stacked hidden layer can provide more useful information to assist the successor task during the inference stage.

In the following, we first introduce the shared semantic representation via learning a GRU attention network. Then, we describe the event detection network where the key issue is the calculation of similarity between news report documents. Next, we describe the event evolution network where the key issue is the calculation of similarity between events. Finally, the training process of the proposed approach is presented in detail.

### 4.1. Shared Semantic Representation

We apply a standard bidirectional gated recurrent unit (GRU) model [44] with an attention mechanism to learn the shared semantic representation across two subtasks. Let *d*=(*w*_1_, *w*_2_,…, *w*_*n*_) be the body words sequence of a news report, where *n* is the news length and *w*_*i*_ is the *i*-th word in the news report. We first map each word *w*_*i*_ into a vector *x*_*i*_ using a pretrained word2vec tool (http://code.google.com/p/word2vec). Specifically, after training using the skip-gram algorithm [45], there is a word embedding matrix; that is, *W*^wrd^ ∈ *ℝ*^*d*^*W*^×|*V*|^, where *V* is the vocabulary of the preset size and *d*^*w*^ is the embedding dimension of word *w*. Then, each word is transformed into a real-valued vector by looking up the pretrained *W*^wrd^; that is, *x*_*i*_=*W*^wrd^*v*^*i*^, where *v*^*i*^ is a one-hot representation of |*V*| dimension. Hence, a news report document can be represented as *X*=(*x*_1_, *x*_2_,…, *x*_*n*_).

Then, we employ a bidirectional gated recurrent unit (GRU) network to extract the hidden vector representation *H*=(*h*_1_, *h*_2_,…, *h*_*n*_) of *X*. The GRU can effectively solve the problem of long-term dependencies in RNN network, and, compared with traditional LSTM, GRU has fewer parameters and is easier to converge; hence, it is more suitable for our real-time system. Specifically, the GRU recurrently processes elements in the sequence *X*. At each step *i*, the hidden state vector *h*_*i*_ of GRU model is updated based on the current vector *x*_*i*_ and the previous state vector *h*_*i*−1_, formulated as *h*_*i*_ = GRU(*x*_*i*_, *h*_*i*−1_), where GRU refers to the standard GRU function [44].

Based on the general GRU representation and following the [33], bidirectional GRU processes the word embedding sequence *X* from both head to tail (⟶) and tail to head (←) and then concatenates two state vectors as output. Formally, it is calculated as follows:

(1) $$\begin{array}{c} {\overset{⟶}{h}}_{i}=\overset{⟶}{\text{GRU}}\left(x_{i},{\overset{⟶}{h}}_{i-1}\right),\\ {\overset{\leftarrow }{h}}_{i}=\overset{\leftarrow }{\text{GRU}}\left(x_{i},{\overset{\leftarrow }{h}}_{i-1}\right),\\ h_{i}=\left[{\overset{⟶}{h}}_{i},{\overset{\leftarrow }{h}}_{i}\right], \end{array}$$

where ${\overset{⟶}{h}}_{i}$ and ${\overset{\leftarrow }{h}}_{i}$ represent the *i*-th state vector generated by GRU from two directions, respectively, *x*_*i*_ is the *i*-th input embedding vector, *h*_*i*_ is the *i*-th hidden state vector, and [*·*, *·*] represents the concatenation operation.

Finally, the attention mechanism [46] is applied to aggregate the hidden vector representation sequence *H*=(*h*_1_, *h*_2_,…, *h*_*n*_) into a fix-length vector as the shared semantic representation for *d*; that is, *H*^*∗*^=Att([*h*_1_, *h*_2_,…, *h*_*n*_]). Specifically, we first map each hidden vector representation *h*_*i*_ into a normalized vector representation *u*_*i*_ in a hidden space; that is, *u*_*i*_=tanh(*W*_*h*_*h*_*i*_+*b*_*h*_), where *W*_*h*_ and *b*_*h*_ are trainable parameters and the value range of *u*_*i*_ is between −1 and 1. Then, an attention weight *α*_*i*_ is computed from the inner product between *u*_*i*_ and a trainable vector *u*_*s*_, followed by a softmax operation; that is,

(2) $$\begin{array}{c} \alpha_{i}=\frac{\exp\left(u_{s}^{T}u_{i}\right)}{\sum_{r}\exp\left(u_{s}^{T}u_{i}\right)}. \end{array}$$

The final output vector is the product between the input hidden vector representation sequence *H*=(*h*_1_, *h*_2_,…, *h*_*n*_) and the learned attention weights *α*=(*α*_1_, *α*_2_,…, *α*_*n*_); that is, *H*^*∗*^=∑_*i*_*α*_*i*_*h*_*i*_. It can be seen that, by assigning different attention weights to each vector representation, important features are emphasized and noisy or irrelevant information is suppressed, so that we can achieve a more discriminative semantic representation of news report documents for subsequent tasks.

### 4.2. Event Detection Network

For the event detection task, our goal is to aggregate news reports related to the same event together [10]. To this end, an online incremental clustering algorithm [27] is employed to cluster incoming news reports into corresponding event groups. Specifically, suppose that we have detected *k* events *E*={*E*_1_, *E*_2_,…, *E*_*k*_}, where each event includes several news reports; that is, *E*_*i*_={*R*_*i*_1__, *R*_*i*_2__,…, *R*_*i*_*n*__}, *i*=1,2,…, *k*. For an incoming news report *R*, we first calculate the similarity score *s*_*i*_*j*__ between it and each new report *R*_*i*_*j*__ in the existing event cluster *E*_*i*_ and take the maximum similarity score *s*_*i*_=max_*j*_{*s*_*i*_*j*__} to measure the similarity between it and the event cluster *E*_*i*_. Then, we consider a threshold *μ* − 3*δ* to decide whether the news report *R* belongs to an existing cluster, where *μ* is the mean of all previous similarity scores and *δ* is the standard deviation. If all similarity scores {*s*_*i*_}, *i*=1,2,…, *k* are below the threshold, we empirically think a new event cluster is detected by including the news report *R*. Otherwise, the news report is added to the most similar existing event cluster, that is, *E*_*m*_, *m*=argmax_*i*_{*s*_*i*_}.

According to the above description, it can be seen that the core of the incremental clustering is to calculate the similarity between two news reports *R*_*i*_ and *R*_*j*_. For this, we consider the Siamese network [5]. Specifically, for two news reports *R*_*i*_ and *R*_*j*_, we first extract their own semantic representation vector *H*_*i*_^*∗*^ and *H*_*j*_^*∗*^, as described in Section 4.1. Then, two vectors are concatenated together, followed by a multilayer perception (mlp) and softmax layer to output the final similarity probability score *P*_dec_, as formulated in

(3) $$\begin{array}{c} H_{\text{dec}}=\sigma \left(W_{\text{dec}}^{h}\left(H_{i}^{*}⊕H_{j}^{*}\right)+b_{\text{dec}}^{h}\right), \end{array}$$

(4) $$\begin{array}{c} P_{\text{dec}}=\text{soft }\max\left(W_{\text{dec}}H_{\text{dec}}+B_{\text{dec}}\right). \end{array}$$

Here, ⊕ denotes the concatenation operation, *σ* represents the sigmoid function, and *W*_dec_^*h*^, *b*_dec_^*h*^, *W*_dec_, and *B*_dec_ are trainable model parameters.

In addition, for the event detection task, the temporal information is also crucial. Hence, we additionally feed the temporal vector *H*_*t*_^*∗*^ of *R*_*i*_ and *R*_*j*_ from the news publish time to the Siamese network, resulting in

(5) $$\begin{array}{c} H_{\text{dec}}=\sigma \left(W_{\text{dec}}^{h}\left(H_{i}^{*}⊕H_{j}^{*}⊕H_{t_{i}}^{*}⊕H_{t_{j}}^{*}\right)+b_{\text{dec}}^{h}\right). \end{array}$$

Here, *H*_*t*_*i*__^*∗*^ and *H*_*t*_*j*__^*∗*^ denote the news publish time vector of *R*_*i*_ and *R*_*j*_, respectively. It is noted that the temporal vector *H*_*t*_^*∗*^ of time *t* is mapped from the Unix timestamp of *t* using the same pretrained word2vec tool as Section 4.1.

The result of event detection network is the detected events set; that is, *E*={*E*_1_, *E*_2_,…, *E*_|*E*|_}, where |*E*| is the number of events in the whole dataset, and *E*_*i*_={*R*_*i*_1__, *R*_*i*_2__,…, *R*_*i*_*n*__}, where *i*_*n*_ is the number of news report documents in the event *E*_*i*_.

### 4.3. Event Evolution Network

After detecting a series of events, we further organize these events into multiple stories in an online manner. Each story covers several interrelated events, which are connected based on their temporal order to characterize the evolving process of that story. Suppose that we have discovered *l* stories *S*={*S*_1_, *S*_2_,…, *S*_*l*_}, where each story includes several detected events, that is, *S*_*i*_={*E*_*i*_1__, *E*_*i*_2__,…, *E*_*i*_*n*__}, *i*=1,2,…, *l*. For an incoming event *E*, our online algorithm to grow the story first identifies the story to which the event belongs. If it does not belong to any existing stories, we create a new story by including it. Afterwards, for all events in each story, we simply connect them by their timestamp order. More specifically, we first calculate the similarity score *δ*_*i*_*j*__ between *E* and each event *E*_*i*_*j*__ in the existing story cluster *S*_*i*_ and further take the maximum similarity score *δ*_*i*_=max_*j*_{*δ*_*i*_*j*__} to measure the similarity between it and the story cluster *S*_*i*_. Then, we still apply a similar threshold strategy to event detection to decide whether the new event *E* belongs to an existing story *S*_*i*_. If the similarity *δ*_*i*_ is below the threshold, a new story is established. Otherwise, the event is added to the most similar existing story group, that is, *S*_*m*_, *m*=argmax_*i*_{*δ*_*i*_}.

Similar to the predecessor event evolution, we still consider a Siamese network to calculate the similarities between events due to its high accuracy and ease of integration, and the input of event evolution network is the event set. Concretely, for two events *E*_*i*_ and *E*_*j*_, we first take the average of semantic representation vectors of news reports which are subordinate to them, as their own semantic representation vectors *ε*_*i*_ and *ε*_*j*_. Then, two vectors and corresponding timestamp vectors are concatenated together and fed into the former event detection network to generate the hidden feature vector *H*_dec_, as formulated in (5).

Furthermore, since the similarity between news reports is highly related to the similarity between corresponding events, for better integration between event detection and event evolution, we additionally feed the obtained hidden feature vector *H*_dec_ of event detection network to the Siamese network, as is formalized in

(6) $$\begin{array}{c} H_{\text{evo}}=\sigma \left(W_{\text{evo}}^{h}\left(\varepsilon_{i}⊕\varepsilon_{j}⊕\varepsilon_{t_{i}}⊕\varepsilon_{t_{j}}⊕H_{\text{dec}}\right)+b_{\text{evo}}^{h}\right), \end{array}$$

(7) $$\begin{array}{c} P_{\text{evo}}=\text{soft}\max\left(W_{\text{evo}}H_{\text{evo}}+B_{\text{evo}}\right), \end{array}$$

where ⊕ denotes the concatenation operation; *σ* represents the sigmoid function; *W*_evo_^*h*^, *b*_evo_^*h*^, *W*_evo_, and *B*_evo_ are model parameters; *ε*_*i*_ or *ε*_*j*_ is semantic representation vector of the event *E*_*i*_ or *E*_*j*_; and *ε*_*t*_*i*__ or *ε*_*t*_*j*__ is the timestamp vector of the event *E*_*i*_ or *E*_*j*_.

Finally, the proposed model generates a series of story sets; that is, *S*={*S*_1_, *S*_2_,…, *S*_|*S*|_}, where |*S*| is the number of stories, and *S*_*i*_={*E*_*i*_1__, *E*_*i*_2__,…, *E*_*i*_*n*__}, where *i*_*n*_ is the number of event from *S*_*i*_. Moreover, events in each story are connected from front to back based on their temporal or logical relationship.

### 4.4. Training and Parameters Setting

The training of the joint model is based on a pair of news reports as input. For the event detection, our training objective is to minimize the cross-entropy loss between the predicted similarity probability and the one-hot ground truth derived from the labeled event ids. Afterwards, we take the *H*_*i*_^*∗*^ and *H*_*j*_^*∗*^ as *ε*_*i*_ and *ε*_*j*_, respectively. And they are fed into the subsequent event evolution network to predict the story similarity. A cross-entropy loss between the predicted similarity probability and the ground truth derived from the labeled story ids is used to optimize the event evolution network and is also propagated to the predecessor event detection network by the hidden feature vector *H*_dec_. Hence, the model parameters from the two networks can be jointly updated using the Nesterov Adam optimizer with the learning rate of 1*e*^−4^. It should be noted that it is reasonable to take the semantic vector of news reports to represent the event for training the event evolution network because an event consists of several related news reports, which should have similar semantic representations; thus, they are close to their average, which is used in the testing.

The model is implemented in Keras with Tensorflow backend. The batch size is set to 16, and the number of epochs is set to 8. Besides, we train word embeddings using the skip-gram algorithm [45] and fine-tune them during the training of the joint model. The size of word embeddings is set to 32, the size of the hidden layers *H*_dec_, *H*_evo_, and shared semantic representation *H*^*∗*^ is set to 16. Before the experiment, we calculated the size of the word bag in advance, and our model predicted 2,500 words, with an extra token covering all the other words. The Dropout technology [47] is used on the word embeddings with a ratio of 0.2 for avoiding overfitting.

## 5. Experiments

In this section, the proposed model is evaluated on a real-world dataset and proved its effectiveness.

### 5.1. Dataset

The proposed JEDE model is evaluated on a real-world Chinese News Report Documents dataset collected by the crawler tool (https://github.com/liuhuanyong/EventMonitor). The original dataset does not contain available labels for all the two subtasks that we investigate. Hence, we have taken efforts to manually annotate 1,931 news report documents with their corresponding *event id* and *story id*, which we from now on refer to as EDENS (Event Detection and Evolution from News Streams) dataset, where the number of stories is 12, the number of events is 694, and each story contains 58 events on average, covering different topics in the open domain, as presented in Table 1. During the annotation of the EDENS dataset, we invited four human annotators, including two doctoral students and two senior undergraduate students majoring in Computer Science and Chinese Language and Literature, to read news reports, respectively, mark news events and event evolution orders artificially, and review the results jointly. The interrater agreement is to refer to Baidu Encyclopedia and Wikipedia.

In addition, we removed common stop words and only kept tokens which are verbs, nouns, or adjectives from these news report documents. In the experiments, 80% of documents with annotated *event id* and *story id* are randomly selected as the training set, and the remaining data serves as the test set.

### 5.2. Evaluation Metrics

We choose several metrics to evaluate the effectiveness of our model for event detection and evolution.

#### 5.2.1. *P*, *R*, and *F*1

The Precision (*P*), Recall (*R*), and *F*1 score are used to evaluate the clustering performance of the event detection and evolution tasks. Formally, they are defined as follows:

(8) $$\begin{array}{c} P=\frac{\left|T\cap T^{\prime }\right|}{\left|T^{\prime }\right|},\\ R=\frac{\left|T\cap T^{\prime }\right|}{\left|T\right|},\\ F1=\frac{2PR}{P+R}, \end{array}$$

where *T*={*E*_1_, *E*_2_,…, *E*_|*E*|_} or {*S*_1_, *S*_2_,…, *S*_|*S*|_} is the set of artificially detected real-world events or stories (ground truth), and *T*′={*E*_1_′, *E*_2_′,…, *E*_|*E*′|_′} or {*S*_1_′, *S*_2_′,…, *S*_|*S*′|_′} is the set of events or stories detected from our model in this paper.

Among the above metrics, Precision measures the percentage of correctly detected events or stories. Recall gives the percentage of events or stories in the ground truth which are correctly detected. *F*-measure is defined as the harmonic mean of Precision and Recall to balance the two metrics. Higher values for Precision, Recall, and *F*1 indicate a better model for event detection and evolution.

#### 5.2.2. Normalized Topic Weighted Minimum Cost (*C*_min_)

For the event detection task, we also take the same evaluation method as in [5] that the normalized Topic Weight Minimum Cost (*C*_min_) from the standard TDT evaluation procedure [10] is used to evaluate clustering accuracy. Formally, it is defined as follows:

(9) $$\begin{array}{c} C_{\min}=\frac{\left(C_{\text{miss}}*N_{\text{miss}}\right)}{\text{len}\left(\text{cluster}\right)}+\frac{\left(C_{\text{fa}}*N_{\text{fa}}\right)}{\text{len}\left(\text{cluster}\right)}, \end{array}$$

where *C*_miss_ and *C*_fa_ are the costs of a missed detection and a false alarm, respectively, and set to *C*_miss_=0.5, *C*_fa_=0.5 in the experiments. In addition, *N*_miss_ and *N*_fa_ are the number of missed detections and false alarms, respectively, and len(cluster) is the size of the event cluster.

From the above formulation, it can be seen that *C*_min_ is a linear combination of missed detection and false alarm error probabilities, which allows a fair comparison between different methods based on such a single metric value. Lower *C*_min_ shows better performance.

#### 5.2.3. ACC

Besides, we use ACC (i.e., accuracy) to evaluate the accuracy of event detection and evolution, which is the ratio of the correctly detected results of our model in all annotated results.

### 5.3. Evaluation of Event Detection Task

We first report the performance of different models for event detection on the Chinese News Report Documents dataset. The following state-of-the-art baseline methods are used for comparisons:

- JEDS [5] is a joint neural model that includes three subtasks: event filtering, detection, and summary, which is specially tailored for Twitter events. It uses shared text representation and neural stacking for joint event detection and summarization. In addition, it uses LSTM as the document representation of each tweet.
- LSH [9] (locality sensitive hashing) is used to detect and track events on unbounded high volume tweet stream in constant time and space, and it utilizes bag-of-words to represent each tweet.
- DBSCAN [31] (Density-Based Spatial Clustering of Applications with Noise) is a classical density-based clustering algorithm. Unlike the *K*-means algorithm, it does not need to preset the number of clusters but can generate clusters for arbitrary shapes based on the number of data speculation clusters.

According to Section 5.2, we use *P*, *R*, *F*1, *C*_min_, and ACC as the evaluation metrics of events detection results, as illustrated in Table 2. We can see that all neural networks based models significantly outperform traditional clustering models by a large margin, which can be explained by the fact that neural network models can capture a richer feature representation compared to discrete models. Among all the methods, our proposed JEDS model yields the best performance, reducing *C*_min_ by around 3%, and improving the F1 score and ACC by around 4% and 1%, respectively, compared to the state-of-the-art method JEDS. This may because our proposed JEDE model uses a more discriminative GRU attention network in place of the regular LSTM in JEDS. Besides, the highest accuracy rate of 77.21% in our model implies that most of the detected document clusters (events) are pure; that is, most events only contain documents that talk about the same real-world breaking news.

### 5.4. Evaluation of Event Evolution Task

Given the set of events extracted by our JEDE model, we further evaluate the performance of the event evolution task on the large twelve stories mentioned above. As described in [8], the event evolution task essentially involves two aspects: (a) identifying the story to which the event belongs and (b) presenting the evolving structure of that story. Therefore, the following experimental results are reported based on these two aspects.

**Figure 3:**
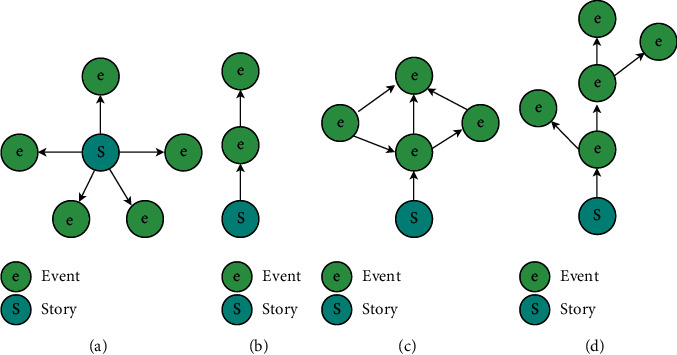
Different structures to characterize a story [8]. (a) Flat structure. (b) Timeline structure. (c) Graph structure. (d) Tree structure.

Specifically, for task (b), following [8], events in a story can be organized in one of the following four structures, as shown in Figure 3:

- Flat structure [10]: this structure does not include the dependencies between events.
- Timeline structure [48]: this structure linearly organizes events by their timestamps.
- Graph structure [3]: this structure checks the connection between all pairs of events and keeps a subset of most strong connections.
- Tree structure [8]: this structure, called story tree, applies a tree to characterize the structures of evolving events within a story. The regular updating operations include merging, extending, and inserting.

To make fair comparisons, we use the same preprocessing and event cluster procedures proposed in our JEDE model to develop different story structures. Specifically, the JEDE models with flat, timeline, graph, and tree structures are termed as JEDE-Flat, JEDE-Timeline, JEDE-Graph, and JEDE-Tree, respectively. In addition, the Story Forest model, which introduces the tree structure (d) in Figure 3, clusters events into stories by calculating the compatibility between events and story tree based on their keyword sets. Therefore, based on the same events clustering results from our JEDE model and event evolving structure, the Story Forest model is used to compare with our proposed JEDE-Tree model on the task (a). Overall, the above five models are used for benchmarking the event evolution task.

We enlisted 50 volunteers to blindly evaluate the results given by different approaches, and, in line with [8], the output story structures are compared from three aspects: the logical coherence of paths (Consistent Paths), the readability of different story structures (Best Structures), and the numbers of one's repeat reading for understanding the story structure (Repeat Readings). In terms of effectiveness, the Consistent Paths and Best Structure are used to evaluate whether a model can help news readers correctly capture the development of events in a short time. In terms of efficiency, the number of repeat readings for understanding the development of a story is recorded in order to compare the efforts a user spent on understanding. As shown in Table 3, in terms of four evolution structures, the tree structure shows the best performance, followed by the timeline structure, which basically accords with human perception. Besides, the flat structure also presents better results than the graph structure, which may indicate that the logic structures of a large portion of real-world news stories are simple, and thus complex graphs are easy to generate an overkill. It should be noted that path coherence is meaningless for flat or graph structure; hence, we ignore the corresponding metric results. A further observation can find that our proposed JEDE-Tree outperforms the Story Forest model on all metrics, with their only difference is in clustering events into a story. This may be explained that our model learns a better event similarity metric by adopting the Siamese neural network models.

### 5.5. Evaluation of Joint Model

In order to investigate whether the joint model improves the accuracy of both tasks in our pipeline setting, we compare our model with several variants, including the following:

- JEDE w/o shared uses a separate bidirectional GRU attention network in event detection and evolution to learn a semantic representation of news reports or events. In this pipeline setting, there is no parameter sharing. The temporal information is not used in learning the similarity metrics of event detection and evolution.
- JEDE w/o stack employs a bidirectional GRU attention network to learn a shared semantic representation *H*^*∗*^ for event detection and evolution without neural stacking and backpropagation between tasks. The temporal information is not used in learning the similarity metrics of event detection and evolution.
- JEDE w/o time uses a shared semantic representation and stacked event detection and evolution. The temporal information is not used in learning the similarity metrics of event detection and evolution.
- JEDE is the proposed Joint Event Detection and Evolution model, which learns a shared semantic representation from a bidirectional GRU attention network for both subtasks and stacks both subtasks with backpropagation training. The temporal information is also used in learning the similarity metrics of event detection and evolution.

Table 4 tabulates the results of different ablation settings, where the performance of task (a) in the event evolution, namely, events clustering into stories, is reported. As can be seen from the table, (1) the original pipeline model (i.e., JEDE w/o shared) shows the worst results but achieves a significant improvement on both tasks by simply sharing the representation of each task (i.e., JEDE w/o stack). (2) Stacking of event detection and evolution is highly beneficial to the joint model, considering backpropagation from event evolution errors to detection (see JEDE w/o time vs. JEDE w/o stack). (3) Removing the input of the temporal information makes the performance of our JEDE model drop on both event detection and event evolution (see JEDE w/o time vs. JEDE). It demonstrates that temporal information can indeed enhance event detection and evolution. In summary, based on the shared semantic representation, neural stacking between both subtasks, and temporal information, the proposed joint model JEDE outperforms all baseline models on event detection and evolution tasks.

### 5.6. Case Study

**Figure 4:**
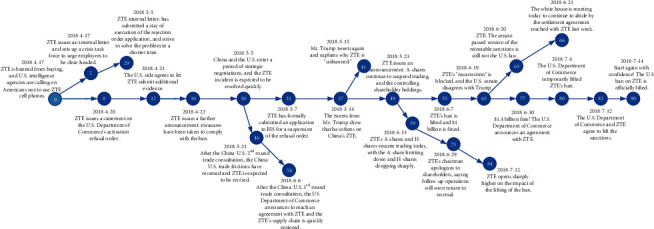
The story tree of “The ZTE incident”.

In this subsection, we provide a qualitative analysis to present the evolution process of events from our model JEDE, taking the story of “The ZTE incident” for example. According to Section 5.4, we select the most effective “Story Tree” structure to represent the event evolution, as shown in Figure 4. The detected story contains 90 nodes, where each node indicates an event in the ZTE incident, and each link represents a temporal or logical connection between two events. For brevity, we randomly delete some nodes for display. Specifically, for instance, event 12 says “The U.S. side agrees to let ZTE submit additional evidence,” and event 55 says “ZTE's ban is lifted and $1 billion is fined.” Most events are arranged by timeline, but there are 5 paths to represent the evolution of events by logical relationship, where the path “0->2->29” talks about the beginning of the ZTE incident, branch “30->46->54” is about the China-US trade consultation and its impact on ZTE, branch “49->59->75->84” is related to the ZTE share price, and so forth. Overall, qualitative results from our model JEDE show that our model successfully aggregates related news reports to the same event and further clearly present the development process of the whole story by the “Story Tree” structure, which demonstrates the effectiveness of our model.

## 6. Conclusions and Future Work

In this paper, we propose an effective neural network model JEDE for Joint Event Detection and Evolution, which benefits both tasks through information sharing between the two tasks. Our model takes the vast streams of trending and breaking news as input and clusters related news reports into several events as well as organizing events in various sensible story structures such as either a tree or a timeline or a flat structure in an online manner. Different from previous popular pipeline settings, our model first uses the bidirectional GRU network and attention mechanism to learn the vectorial semantic representation of news reports as well as events, without the bag-of-words assumption, which is globally shared on both event detection and evolution tasks. Then, two similarity metrics about a pair of news documents or events are learned continuously by neural stacking, so that information is better shared between the predecessor event detection and successor event evolution networks. Empirical experiments on a newly annotated real-world dataset EDENS demonstrate the superior performance of our model over several baseline models on both subtasks. In summary, our model is able to effectively deal with event detection and evolution online for massive amounts of breaking news data.

Despite the remarkable improvements, the proposed model still faces some challenges. For example, as we all know, event description is crucial in event mining task. However, our model just simply uses the title of the earliest news report to summary an event, but without considering the richer contextual information contained in the clustered news reports. Hence, a well designed summarization module is necessary in the future joint model. Besides, this paper primarily talks about events about formal news media, and it is implicitly assumed that detected events are all realistic, due to the authority of formal news reports. However, for emerging social media, lack of supervision and free Twitter expressions may produce some rumor events, which may hinder people's understanding of the truth about topics of interest [49, 50]. This issue has not yet been solved by the proposed model; hence, it is worth thinking deeply about how to detect and exclude the interference of rumors and present a clean and clear event evolution process for informal social media. The rumor propagation [51–53] mechanism is also another research topic of interest in the future.

## Figures and Tables

**Table 1:** The details of our EDENS dataset.

ID	Story	Document	Event	Time
1	The ZTE incident	474	108	2018/4/17–2018/7/15
2	Zhang Yingying's disappearance	201	87	2017/6/8–2018/6/11
3	Yulin pregnant woman jumps to death	55	29	2017/8/31–2017/11/28
4	The Wei Zexi incident	322	137	2016/5/1–2018/5/22
5	Home Inns hotel attack	105	37	2016/4/6–2016/11/5
6	The Luo Yixiao donation incident	151	51	2016/11/30–2018/1/22
7	The Lei Yang whoring incident	31	13	2016/5/10–2016/12/24
8	Kindergarten child abuse	101	30	2016/10/28–2018/5/30
9	Hangzhou babysitter arson	326	103	2017/6/22–2018/6/4
10	Xu Yuyu Telecommunications fraud	62	39	2016/8/25–2018/2/13
11	Yu Huan kills the mother abuser	31	20	2017/3/26–2018/5/8
12	The tiger biting incident	64	40	2016/7/25–2017/12/21

**Table 2:** Comparison of different models on event detection.

Method	*P* (%)	*R* (%)	*F*1 (%)	*C*_min_ (%)	ACC (%)
DBSCAN	51.39	70.49	59.44	72.57	51.71
LSH	59.31	61.06	60.17	69.47	57.92
JEDS	67.44	71.19	69.26	53.20	76.08
**JEDE**	**75.14**	**71.75**	**73.41**	**50.06**	**77.21**

The bold values highlight the performance of the proposed JEDE model on several metrics. It can be seen from Table 2 that our model performs better than other baseline models. We have described these results in Section 5.3.

**Table 3:** Results of event evolution about different story structure generation algorithms.

Method	Consistent Paths	Best Structures	Repeat Readings
JEDE-Graph	—	6.1784	15
JEDE-Flat	—	6.2996	12
JEDE-Timeline	7.5004	7.1860	12
Story Forest	7.8244	7.2706	11
**JEDE-Tree**	**8.1106**	**7.8626**	**8**

The bold values highlight the performance of the proposed JEDE-Tree model on several metrics. It can be seen from Table 3 that our model with the tree structure performs better than other baseline models. We have described these results in Section 5.4.

**Table 4:** Comparison of our model with variants.

Task	Event detection					Event evolution			
Model	*P* (%)	*R* (%)	*F*1 (%)	*C*_min_ (%)	ACC (%)	*P* (%)	*R* (%)	*F*1 (%)	ACC (%)
JEDE w/o shared	53.73	59.42	56.43	68.02	55.23	56.09	64.74	60.11	53.98
JEDE w/o stack	57.57	63.83	60.54	67.35	56.39	63.52	64.68	64.09	68.06
JEDE w/o time	68.36	70.19	69.26	51.11	64.94	69.41	70.75	70.07	70.04

**JEDE**	**75.14**	**71.75**	**73.41**	**50.06**	**77.21**	**73.83**	**68.63**	**71.14**	**72.18**

The bold values highlight the performance of the proposed JEDE model on several metrics. It can be seen from this ablation study that shared semantic representation, neural stacking, and time information help the proposed JEDE model achieve the optimal performance. We have described these results in Section 5.5.

## Data Availability

The data used to support the findings of this study are available from the corresponding author upon request.
